# Follow-up of newborns treated with extracorporeal membrane oxygenation: a nationwide evaluation at 5 years of age

**DOI:** 10.1186/cc5039

**Published:** 2006-09-11

**Authors:** Manon N Hanekamp, Petra Mazer, Monique HM van der Cammen-van Zijp, Boudien JM van Kessel-Feddema, Maria WG Nijhuis-van der Sanden, Simone Knuijt, Jessica LA Zegers-Verstraeten, Saskia J Gischler, Dick Tibboel, Louis AA Kollée

**Affiliations:** 1 Department of Pediatric Surgery, Erasmus Medical Centre, Sophia Children's Hospital. Rotterdam, The Netherlands; 2 Department of Physical Therapy, Subdivision Peadiatric Physiotherapy, Erasmus Medical Centre, Sophia Children's Hospital, Rotterdam, The Netherland; 3 Department of Medical Psychology, Radboud University Nijmegen Medical Centre, The Netherlands; 4 Department of Paediatric Physical Therapy, Radboud University Nijmegen Medical Centre, The Netherlands; 5 Department of Allied Health Care, Speech and Language Pathology, Radboud University Nijmegen Medical Centre, The Netherlands; 6 Department of Neonatology, Radboud University Nijmegen Medical Centre, The Netherlands

## Abstract

**Introduction:**

Extracorporeal membrane oxygenation (ECMO) is a supportive cardiopulmonary bypass technique for babies with acute reversible cardiorespiratory failure. We assessed morbidity in ECMO survivors at the age of five years, when they start primary school and major decisions for their school careers must be made.

**Methods:**

Five-year-old neonatal venoarterial-ECMO survivors from the two designated ECMO centres in The Netherlands (Erasmus MC – Sophia Children's Hospital in Rotterdam, and University Medical Center Nijmegen) were assessed within the framework of an extensive follow-up programme. The protocol included medical assessment, neuromotor assessment, and psychological assessment by means of parent and teacher questionnaires.

**Results:**

Seventeen of the 98 children included in the analysis (17%) were found to have neurological deficits. Six of those 17 (6% of the total) showed major disability. Two of those six children had a chromosomal abnormality. Three were mentally retarded and profoundly impaired. The sixth child had a right-sided hemiplegia. These six children did not undergo neuromotor assessment. Twenty-four of the remaining 92 children (26%) showed motor difficulties: 15% actually had a motor problem and 11% were at risk for this. Cognitive delay was identified in 11 children (14%). The mean IQ score was within the normal range (IQ = 100.5).

**Conclusion:**

Neonatal ECMO in The Netherlands was found to be associated with considerable morbidity at five years of age. It appeared feasible to have as many as 87% of survivors participate in follow-up assessment, due to cooperation between two centres and small travelling distances. Objective evaluation of the long-term morbidity associated with the application of this highly invasive technology in the immediate neonatal period requires an interdisciplinary follow-up programme with nationwide consensus on timing and actual testing protocol.

## Introduction

Extracorporeal membrane oxygenation (ECMO) is a cardiopulmonary bypass technique for providing life support in acute reversible cardiorespiratory failure when conventional management is not successful. Most patients receiving ECMO support are neonates suffering from persistent pulmonary hypertension of the newborn, primary or secondary to meconium aspiration syndrome, sepsis, or congenital diaphragmatic hernia. Worldwide, over 18,700 neonates have been treated with ECMO for respiratory problems, and the overall survival rate was 77% [1].

The UK Collaborative ECMO Trial Group in 1996 presented the results of a randomised controlled clinical trial, showing a significant survival benefit of ECMO, without a concomitant rise in severe disability at one year of age [2,3]. Even for the 35 neonates with congenital diaphragmatic hernia (CDH) the risk of death was reduced (relative risk, 0.72; 95% confidence interval, 0.54–0.06; *P *= 0.03). Of the 18 neonates with CDH allocated to ECMO, however, 14 died (one after discharge) and only three children survived to age 4 years. All 17 infants in the conventional management arm died before discharge [4]. No other therapeutic intervention (that is to say, high-frequency oscillatory ventilation, surfactant, and inhaled nitric oxide) for neonatal acute respiratory failure has such a positive impact on mortality and morbidity [5].

Nevertheless, the severity of illness of potential candidates for ECMO, as well as the risks associated with the procedure itself, places the ECMO survivor at high risk of developing brain injury and subsequent function deficits. All patients receiving ECMO support have suffered from severe respiratory failure prior to treatment. Prolonged episodes of severe hypoxaemia may occur, despite the administration of 100% oxygen. The inevitable high ventilatory pressures and hyperventilation may cause alterations in cerebral blood flow [6,7]. In venoarterial ECMO the right common carotid artery and right internal jugular vein are cannulated and subsequently ligated after bypass is finished. Finally, the heparin that is administered to prevent the blood from clotting might cause intracranial haemorrhage as a confounder for long-term morbidity. It is not easy, therefore, to predict the long-term outcome of neonates treated with ECMO.

The few reports on structural follow-up of ECMO survivors either describe infants up to age 2 or patients from a single centre with wide age distribution [3,8-15]. The reports point out that logistic problems may prevent patients from being available for predetermined, structural evaluation. Major disabilities in terms of severe developmental delay or neuromotor disabilities were reported in some 20% of ECMO survivors [3,8,10,12]. The range of morbidity widens with evaluation after age one, when assessment of cognitive skills, coordination, behavioural difficulties, and sensory loss can be more precise [16]. Long-term longitudinal follow-up of these children therefore seems essential for placing ECMO results in perspective. Only two studies describe longitudinal neurodevelopmental evaluation at school age [16,17]. Although surviving children treated for severe life-threatening respiratory failure soon after birth show considerable long-term morbidity, the results of the UK ECMO trial point to a favourable profile of long-term morbidity in the group assigned to ECMO [16].

Glass and colleagues reported a 61% response rate; 25% did not participate because of the long travelling distances in the USA [17]. Neonatal ECMO in The Netherlands is provided in two designated centres only, authorised by the Dutch government. All parents of ECMO survivors are invited to enter their child into a redesigned follow-up programme. High response rates are feasible because travelling distances are short in The Netherlands and regionalised high-risk perinatal care, including ECMO, is available. The children are scheduled to undergo assessment at ages 6, 12, 18 and 24 months and 5, 8 and 12 years. In the present paper we present the follow-up findings at age 5 years, when children are in the first year or second year of primary school and major decisions for their future school careers must be made.

## Materials and methods

### Patients

The study population included five-year-old neonatal venoarterial ECMO survivors from both ECMO centres in The Netherlands (the Erasmus MC – Sophia Children's Hospital Rotterdam and University Medical Center Nijmegen). The patients were seen either between May 2001 and December 2003 (Rotterdam) or between March 1998 and December 2003 (Nijmegen). According to national consensus on neonatal follow-up and the obligation to provide these data based on reports of the Dutch Ministry of Health, the assessment protocol is the standard of care in The Netherlands following ECMO. As a consequence IRB approval was waived, while all parents were routinely informed about the long-term follow-up programme in the neonatal period of life of their child.

### Assessment protocol

Complete assessment included a one hour medical assessment by a paediatrician/neonatologist experienced in the follow-up evaluation, a 1.5-hour neuromotor assessment by a paediatric physiotherapist, and a three hour neuropsychological assessment by a psychologist or psychological test assistant (Table 1). In Nijmegen a speech therapist assessed speech and language development, and in Rotterdam the psychologist performed the neuropsychological assessment.

The complete assessment took place in one day. The sequence of the different assessments could vary for logistic reasons.

In addition, one month before assessment, the parents were invited to complete questionnaires on parental socio-economic status and the child's current general health and behaviour.

Perinatal characteristics such as birthweight, gestational age, age at start of ECMO, duration of ECMO, primary diagnosis, and possible intracranial abnormalities were obtained from each centre's ECMO registry and are included in the Extracorporeal Life Support Organisation Registry Report [1].

### Medical assessment

Medical assessment consisted of taking the child's medical history, the measurement of growth parameters, and a standard physical examination followed by a standard neurological examination. The length and weight were expressed as the standard deviation (SD) score using the Dutch Growth Analyser, version 2.0 (Dutch Growth Foundation, Rotterdam, Netherlands). The results of the neurological examination were categorised into normal (no neurological abnormalities), minor neurological dysfunction (neurological abnormalities without influence on normal posture or movement), and major neurological dysfunction (neurological abnormalities with abnormal posture or movements, including seizure disorders).

#### Neuromotor assessment

The Movement Assessment Battery for Children (M-ABC) was used to measure motor functioning [18]. A Dutch standardisation study has shown that the original norm scores and cut off points can also be applied to Dutch children. Good validity and reliability have been demonstrated [18,19].

The M-ABC was developed for children aged 4–12 years. The measure has four age-related item sets, each consisting of eight items: three manual dexterity items (a time-related task for each hand separately, a bimanual coordination task, and a graphical task with the preferred hand), two ball skill items (a task of catching a moving object and a task of aiming at a goal), and three balance items (static balance, dynamic balance while moving fast, and dynamic balance while moving slowly). Scores may range from 0 to 5 for each item. A high score on the M-ABC indicates poor performance. The total impairment score, which is the sum of the item scores, was calculated as a percentile score. A score below the 5th percentile is indicative of a motor problem, a score between the 5th and 15th percentile means borderline performance, and a score above the 15th percentile is a normal score [18].

##### Exercise test

The children seen in Rotterdam performed a graded, maximum exercise test using a motor-driven treadmill. The treadmill was programmed for increases in angle of inclination and speed every three minutes according to the Bruce protocol [20,21]. The Bruce protocol starts with a speed of 2.7 km/hour at an incline of 10%. The children are encouraged to perform to voluntary exhaustion. The maximal endurance time was used as criterion of exercise capacity and compared with data reported previously [21,22].

#### Neuropsychological assessment

##### Cognitive development

A short version of the Revised Amsterdam Intelligence Test (RAKIT) for children was used to evaluate cognitive development. The RAKIT is a well-known standardised instrument in The Netherlands for children aged 4–11 years. Good reliability and validity have been demonstrated [23,24]. The short version contains six subtests. The raw subtest scores are converted into standardised scores, which are then transformed into a short RAKIT intelligence quotient (IQ) with a mean of 100 and a SD of 15. Cognitive delay was defined by a test result more than -1 SD below the norm (that is to say, IQ ≤ 85).

##### Visual–motor integration

The Developmental Test of Visual–Motor Integration for children aged from 3 to 18 years measures the integration of visual perceptual and motor abilities [25]. Children are asked to copy figures of increasing geometric complexity. The computed raw item scores are transformed into a visual–motor integration standard score with a mean of 100 and a SD of 15.

##### Behaviour

The Dutch versions of the Child Behaviour Checklist and the Teacher's Report Form were completed by parents and teachers, respectively [26,27]. Both have been standardised for the Dutch population from 4 to 18 years old, and rate 120 problem behaviour items on a three-point scale (0 = not true, 1 = somewhat true or sometimes true, 2 = very true or often true) [28,29]. A total problem score is computed by summing the scores of all items. Two broadband scales were constructed: an internalising scale including withdrawn behaviour, somatic complaints without physical cause, and anxious-depressive feelings; and an externalising scale including aggressive and delinquent behaviour. Total scores ≥60 classify children in the borderline/clinical range.

##### Language development

Language development was assessed with the Reynell Test and the Schlichting Test. The Reynell test assesses receptive language development of Dutch-speaking children between ages 1 and 6 years [30]. Expressive language is not required since the children may respond nonverbally.

The Schlichting Test assesses language expression of Dutch-speaking children between ages 1 and 6 years [31]. Two subtests were applied: one testing knowledge of grammatical structure (syntactical development), and the other subtest measuring active vocabulary (lexical development).

The numbers of correct answers in the tests were transformed into standard quotient scores with a mean of 100 and a SD of 15. The following categories were discerned: delayed/abnormal development (score less than -2 SD), at risk (score from -1 SD to -2 SD), and normal (score greater than -1 SD).

### Data analysis

Data are presented for the entire group and also by diagnosis. An independent-sample Student *t *test was performed when appropriate to analyse differences between the study group and general population norms. *P *< 0.05 represented statistical significance.

A chi-square test was performed to test whether the motor performance scores in this ECMO population differed significantly from the distribution in the normal population. *P *< 0.05 was considered statistically significant.

## Results

**Figure 1:**
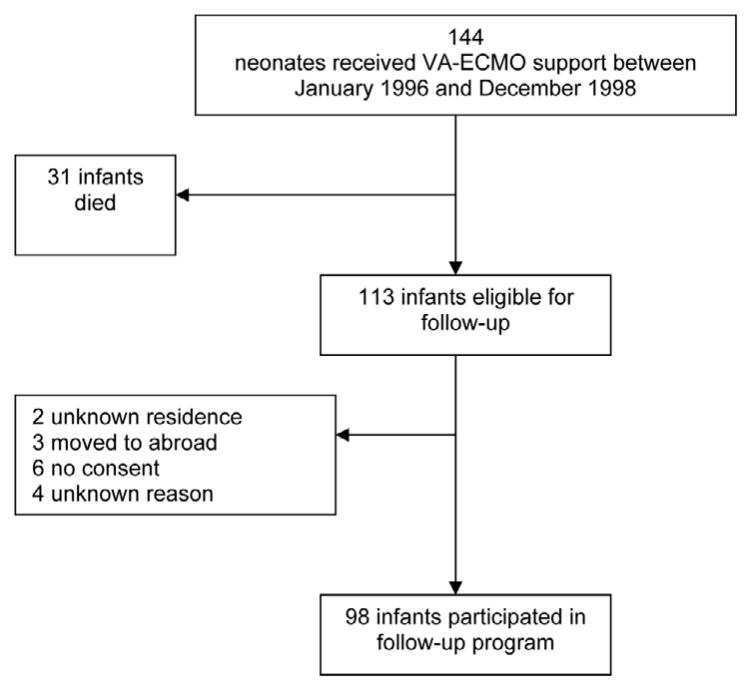
Flowsheet of infants included in the follow-up programme. VA-EMCO, venoarterial extracorporeal membrane oxygenation.

A total of 144 neonates received venoarterial-ECMO support from January 1996 up to and including December 1998. Thirty-one of them (22%) died before age 5 years, all during first admission at median age 21 days (interquartile range, 11–35 days; range, 2–120 days). Fourteen infants were lost to follow-up for various reasons. The present addresses of two children could not be traced, the families of three children moved abroad, and parental consent was withheld for five children. Four other children failed to appear, even after repeated invitations. Ninety-nine infants therefore participated in the follow-up programme (Figure 1). The parents of one child, however, withheld consent to use data for publication purposes, so eventually we present data of 98 children (35 children in Rotterdam, 63 children in Nijmegen) (87% of all survivors).

The perinatal characteristics and ECMO-treatment characteristics of the participants are presented in Table 2. The children's basic characteristics at time of follow-up are presented in Table 3.

### Outcome medical assessment

Seventeen children (17%) were found to have a neurological disorder. Six of those (6%) showed major neurodevelopmental disability, including two children with a chromosomal abnormality. Of the latter, one child was known to have Down syndrome and the second child (diagnosed with CDH) showed unbalanced translocation of chromosome 11–22 (unknown at the time of ECMO). This boy was severely impaired and mentally retarded, and is known to have died at age six years.

Of the other four children with major neurological disorder, one had a right-sided hemiplegia caused by nonhaemorrhagic infarction during ECMO. He walked with an orthesis and attended special education. The second child had developed a right-sided hemiplegia as a result of left-sided cerebral hemiatrophy. He was confined to a wheelchair and was mentally retarded. The third child with major neurological disorder (diagnosed with meconium aspiration syndrome) had severe asphyxia and had been resuscitated in the immediate postnatal period. Still suffering from a seizure disorder, she used a walking frame, and she was mentally retarded. The fourth child suffered from seizures, used a wheelchair, and was mentally retarded.

Eleven children (11%) showed minor neurological dysfunction, varying from strength differences in the upper and lower extremities to very mild hemiplegia and a mild form of West syndrome (one child).

The mean (SD score) weight and height for the entire population were -0.5 (1.5) and -0.4 (1.2), respectively (Table 3). Both parameters were significantly below zero (*P *= 0.001 and *P *= 0.002, respectively).

Eighteen children (18%) had respiratory complaints. Twelve of them regularly used a combination of β-sympathicomimetic drugs and inhalation steroids. None of the children needed supplemental oxygen. Two of the total population were followed because of a muscular ventricular septal defect, without haemodynamic consequences; one because of atrial septal defect. One of the 20 children diagnosed with CDH was still on (nightly) tube feeding because of low weight (-3.4 SD) and pulmonary problems, and a second child had received tube feeding until his fourth birthday. The child who was known with unbalanced translocation of chromosome 11–22 was fed through a gastrostomy drain and had undergone a Nissen fundoplication because of gastrooesophageal reflux. Another child, not diagnosed with CDH, was also fed through a gastrostomy drain.

### Outcome neuromotor assessment

Excluding the six children with major neurodevelopmental disability, 92 of the 98 children were tested using the M-ABC. Twenty-four children (26.1%) were classified as having some kind of motor difficulty (percentile score < P 15), which represents a significantly higher proportion than expected (chi-square test, *P *< 0.005).

Fourteen children (15.2%) had scores indicative of a motor problem (percentile score < P 5) (chi-square test, *P *< 0.001), 10 children (10.9%) had borderline performance (percentile score < P 15 but > P 5), and 68 children (73.9%) performed normally (percentile score > P 15) (Table 4).

A comparison with population norms revealed that the mean (SD) M-ABC score of the total group was significantly below the reference value: 8.4 (8.1) versus 5.2 (5.6) (*P *< 0.001) [18].

Twenty-nine of the 35 children seen in Rotterdam performed the exercise test according to the Bruce protocol (Table 5). Five children with major neurological impairment could not perform the test. One child (diagnosed with CDH) was too anxious to use the treadmill and performed a six-minute walking test instead. The height and weight of the 29 children (15 boys) were expressed as SD scores. These were not significantly below or above the reference value (SD = 0) and there were no significant differences between boys and girls. Comparison of endurance times with the Canadian norms reported by Cumming and colleagues [21] revealed a significantly lower mean (SD) endurance time for the boys: 9.0 (1.2) versus 10.4 (1.9) (*P *< 0.005). The mean endurance time for the girls was not significantly different: 9.2 (1.8) versus 9.5 (1.8) (*P *= 0.6).

### Outcome neuropsychological assessment

To create a mutually comparable group, three children with chromosomal or syndromal abnormalities as well as 11 children who did not speak Dutch as their native language and one child with severe hearing problems were excluded from data analysis. One child's data on all neuropsychological tests were lost, leaving 82 children for analysis. For three children all data on cognitive development were missing. Three children could not be successfully tested on expressive language, and one child could not be tested either on expressive or on receptive language. Visual–motor integration in Rotterdam was tested in 28 out of 35 children. Major neurological impairment precluded testing in five children and the data of two other children were missing. The neuropsychological outcome data are presented in Table 6.

#### Cognitive development

Eleven children (14.1%) showed cognitive delay. The mean RAKIT score of the total group (IQ = 100.5) did not differ significantly from the Dutch norm.

#### Language development

In the expressive language test, 13 children (16.7%) scored ≥ 1 SD below the norm on grammar and vocabulary. In the receptive language test, six children (7.3%) scored ≥ 1 SD below the norm. The mean scores on grammar and receptive language were significantly above the Dutch norm.

#### Visual–motor integration

Seven children (25%) scored ≥ 1 SD below the norm. The mean score of the total group did not differ significantly from the Dutch norm.

#### Behaviour

The results of the Child Behaviour Checklist are presented in Table 7. Of all children, 12.8% had a total problem score above 63, indicating behavioural problems. Internalising problems occurred slightly more than did externalising problems.

## Discussion

The present report presents nationwide neurodevelopmental sequelae of 98 venoarterial-ECMO-treated neonates at age 5 years (87% of all survivors). Seventeen children (17%) presented with major or minor neurological disorders. Another 24 children (26.1%) of the children who participated in the neuromotor assessment presented with some kind of motor difficulty, 14 of whom (15.2%) had an actual motor problem and 10 of whom (10.9%) were at risk for a motor problem. Cognitive delays were identified in 11 children (14% of 82 analysed children).

Two of the 17 children with neurological disability had a chromosomal disorder accounting for neurological impairment, and one child had West syndrome associated with mental retardation and seizures. Four of the remaining 14 patients (14%) had major neurological impairment and 10 children had minor neurological impairment without an underlying disorder. Our findings seem not completely consistent with findings reported by Glass and colleagues [17] in 103 children: 17% of children in that study had one or more major disability versus 14% in our group. Glass and colleagues, however, ranked mental disability, as well as motor disability and seizure disorders, also under major disability. Had we included children who scored abnormal in the medical assessment, motor assessment, or mental assessment as well, we would have found a similar proportion (17%).

The UK ECMO Trial Group has reported on the outcome of ECMO-treated neonates at age 4 years [16]. A consistent comparison is hampered by the fact that methods were different. In the United Kingdom one paediatrician assessed the children in six clinical domains, including cognitive ability, neuromotor skills, general health, behaviour, vision, and hearing. Nineteen per cent of the children had test scores outside the normal range. With regard to 'disability', 13% of the children were moderately to severely disabled, which is consistent with the 14% we report.

The rate of motor difficulties in our cohort was 26% (score < P 15); 15% of the children had an abnormal motor score (score < P 5). This 15% we found exceeds the 6% reported by Glass and colleagues [17]. Unfortunately, few follow-up studies have used standardised tests such as the M-ABC to assess neuromotor outcome. Even in our study some of the children with minor motor difficulties were assessed normal at neurological examination. In the M-ABC assessment, however, the children are stressed to move under velocity or accuracy demands. Such circumstances are more sensitive to detect motor performance problems. It is essential, therefore, that professionals with specific experience should assess the developmental domains in the context of a structured follow-up programme.

In our study 29 children performed a maximum exercise test. The maximal endurance time was used as the criterion of exercise capacity, and we compared outcomes with the data presented by Cumming and colleagues [21]. Binkhorst and colleagues in 1992 published references values for normal exercise performance in Dutch boys and girls aged 4–18 years using the Bruce treadmill protocol [22]. The authors included few 4 year olds and the number of 6 year olds is unclear, however, and they did not provide means and SDs for these ages. This is why we did not use these Dutch reference values. Nevertheless, 41% of the children in our study would score below the 5th percentile according to these Dutch reference values. The question is whether this can be explained by impaired physical condition of ECMO-treated patients or by the fact that the reference values established by Binkhorst and colleagues insufficiently reflect the exercise performance of contemporary healthy Dutch children. Future studies are needed and will be performed in Erasmus MC – Sophia Children's Hospital in the near future.

Follow-up at age 5 is important because children are in their first year or second year of primary school, at the start of their further school career. Eleven children (14%) showed cognitive delay, a proportion comparable with that reported by Glass and colleagues (13%). The IQ summary scores are comparable as well: 100 in our cohort versus 96 in their study. Although in the UK ECMO trial cognitive ability at age 4 did not show evidence of a difference between the two trial groups, 23% of ECMO-treated children showed cognitive delay (defined as IQ greater than -1 SD) [16].

Behavioural problems beyond the clinical cutoff point were identified in 11 children in our cohort. These problems might contribute to school failure, even in the absence of cognitive delay [32].

Language development scores were all above population norms. Children without Dutch as their native language who had difficulty understanding and speaking Dutch were, however, excluded from these tests. Still, language development seems unaffected.

In the absence of a matched control group it remains difficult to establish to what extent ECMO treatment contributes to the outcome. The UK ECMO trial did show a benefit of ECMO based on the primary outcome of death or severe disability. Children were assessed at age 4 in six different domains (cognitive ability, neuromotor skills, general health, behaviour, vision, and hearing). The trial defined outcome as normal, impaired, or disabled on the basis of the degree of functional loss in any of the domains. There was no evidence of significant difference regarding cognitive ability and motor disability between the conventional treatment and ECMO groups. The overall rate of moderate disability in the conventional group was 11% versus 13% in the ECMO group. Severe disability was only reported in the ECMO group (that is to say, 3%) [16]. The rate of disability (that is to say, cerebral palsy) reported in a study of 89 surviving children with moderate to severe perinatal asphyxia at age 8 years was 15%. Ten per cent of children had profound cognitive delay [33]. The intelligence quotient in a group of nondisabled children with mild and moderate perinatal asphyxia was 106 (± 14) [33]. These proportions are in the same range as the proportions reported in the present study.

Since all infants received venoarterial ECMO, would venovenous ECMO improve cognitive or neuromotor outcome? When using the Extracorporeal Life Support Organisation Registry [1], no significant difference in primary outcome between venovenous ECMO and venoarterial ECMO has been reported [34].

## Conclusion

The outcome figures of ECMO-treated neonates at follow-up at age 5 years presented in the present study show considerable morbidity, but they do not greatly differ from those reported in previous publications on ECMO-treated neonates [16,17]. The high response rate of 87% (versus 61% by Glass and colleagues [17]) was feasible for various reasons: cooperation between two centres, small travelling distances, as well as the quality of the health care system in The Netherlands. We believe that a successful follow-up programme of severely ill neonates should be structured in consultation with representatives from different disciplines, such as a paediatrician, a paediatric physiotherapist, a psychologist, and a speech therapist. Further longitudinal follow-up studies will focus on the relationship between neonatal status and test results at 5 years and on detailed analysis of the different domains. Within the framework of the nationwide follow-up programme, longitudinal data at ages 8 and 12 years are expected to become available in due time.

## Key messages

• Follow-up after neonatal venoarterial ECMO in children at age 5 showed 17% of children with major or minor neurological disorders and 26% with some kind of motor difficulty.

• Cognitive delay was present in 14% of the 5-year-old children after neonatal venoarterial ECMO.

• A successful follow-up programme of severely ill neonates should be structured in consultation with representatives from different disciplines.

## Abbreviations

CDH = congenital diaphragmatic hernia; ECMO = extracorporeal membrane oxygenation; IQ = intelligence quotient; M-ABC = Movement Assessment Battery for Children; RAKIT = Revised Amsterdam Intelligence Test; SD = standard deviation.

## Competing interests

The authors declare that they have no competing interests.

## Authors' contributions

MNH participated in the follow-up programme as a medical doctor and drafted the manuscript. PM participated in the follow-up programme in Rotterdam as a psychologist and helped to draft the manuscript. MHMvdC-vZ participated in the follow-up programme in Rotterdam as a paediatric physiotherapist. BJMvK-F participated in the follow-up programme in Nijmegen as a psychologist. MWGN-vdS participated in the follow-up programme in Nijmegen as a paediatric physiotherapist. SK participated in the follow-up programme in Nijmegen as a speech therapist. JLAZ-V participated in the data analysis. SJG participated in the follow-up programme as a paediatrician. DT participated in the coordination and design of the study and helped to draft the manuscript. LAAK participated in the coordination and design of the study and helped to draft the manuscript. All authors read and approved the final manuscript.

## Figures and Tables

**Table 1:** Assessment protocol at 5 years of age

	Time (hours)	Instrument	Nijmegen	Rotterdam
Medical assessment (paediatrician)	1	Physical and neurological examination	X	X
Neuromotor assessment (paediatric physiotherapist)	1.5	Movement Assessment Battery for Children	X	X
		Exercise test		X
Neuropsychological assessment (psychologist and speech therapist)	3			
Intelligence		Revised Amsterdam Intelligence Test/Wechsler Intelligence Scale for Children	X	X
Visual – motor integration		Beery		X
Receptive language development		Reynell	X	X
Expressive language development		Schlichting	X	X
Behaviour		Child Behaviour Checklist/Teacher's Report Form	X	X

**Table 2:** Perinatal and extracorporeal membrane oxygenation (ECMO) characteristics

Male/female	60/38
Birthweight (kg)	3.3 (2.9–3.8)
Gestational age (weeks)	40 (38–41)
Apgar score at 1 minute/5 minutes	5/7
Primary diagnosis	
Meconium aspiration syndrome	51
Congenital diaphragmatic hernia	20
Sepsis	11
Persistent pulmonary hypertension of the newborn	15
Congenital cystic adenoid malformation of the lung	1
Outborn (*n*)	
Home	10
>20 km from ECMO centre	51
<20 km from ECMO centre	30
Oxygenation index prior to ECMO^a^	39 (24–58)
Alveolar arterial oxygen distention gradient^b^	622 (606–637)
Age at start of ECMO (hours)	28 (17–43)
Duration of ECMO support (hours)	155 (127–188)
Duration of mechanical ventilation (days)	16 (13–22)
Supplemental oxygen after ECMO (days)	8 (4–16)
Duration of first admission (days)	38 (30–55)
Haemorrhagic intracranial abnormalities (*n*)	
Minor: intraventricular haemorrhage grade 1 and grade 2	8
Major: intraventricular haemorrhage grade 3 and grade 4	0
Nonhaemorrhagic intracranial abnormalities (*n*)	
Minor: ventricular dilatation and focal atrophy	17
Major: general atrophy and infarcts	4
Observed infants with epileptic insults (*n*)	27
Patients treated with phenobarbital as prophylaxis (*n*)	37
Duration of phenobarbital treatment in infants with epileptic insults (days)	49 (21–90)

Perinatal characteristics of the 98 children available for analysis, presented as *n *(%) of infants or median (interquartile range). ^a^Calculated as [(mean airway pressure × FiO_2_)/PaO_2_] × 100. ^b^Calculated as P_atm _– P_H2O _– PaO_2 _– PaCO_2 _(PaO_2 _and PaCO_2 _in mmHg).

**Table 3:** Basic characteristics of the study group at 5 years of age

	Total group (*n *= 98)
Males/females	60/38
Age (months)	62 (3.0)
Weight SD score	-0.5 (1.5)*
Height SD score	-0.4 (1.2)**
Weight for height SD score	-0.4 (1.4)***
Socioeconomic status (%)	
High	26 (27)
Normal	49 (50)
Low	19 (19)
Unknown	4 (4)
Ethnic group (%)	
White	85 (87)
African	3 (3)
Asian	1 (1)
Turkish or Moroccan	9 (9)

Data presented as *n *(%) of patients or mean (standard deviation (SD)). The mean weight, height and weight for height (SD scores) for the entire population were all significantly below zero: * *P *= 0.001, ** *P *= 0.002, *** *P *= 0.008. Children with congenital diaphragmatic hernia had significantly lower height and weight than children with meconium aspiration syndrome (*P *< 0.001).

**Table 4:** MovementAssessment Battery for Children results

	Total group (*n *= 92)	Meconium aspiration syndrome (*n *= 49)	Congenital diaphragmatic hernia (*n *= 19)	Sepsis (*n *= 11)	Persistent pulmonary hypertension of the newborn (*n *= 12)
Total impairment score (mean (standard deviation))	8.4 (8.1)*	6.8 (6.6)	13.4 (10.3)	7.5 (7.5)	7 (7.1)
Score < P 5, motor problem (*n *(%))	14 (15.2%)**	3 (6.1)	7 (36.8)	1 (9.1)	2 (16.7)
Score P 5–P 15, borderline (*n *(%))	10 (10.9%)	3 (6.1)	4 (21.1)	2 (18.2)	1 (8.3)
Score > P 15, normal (*n *(%))	68 (73.9%)***	43 (87.8)	8 (42.1)	8 (72.7)	9 (75)

*t test: significant, *P *< 0.001. ** Chi-square test: significant, *P *< 0.001. ***Chi-square test: significant, *P *< 0.005.

**Table 5:** Exercise test

	Total group (*n *= 29)	Boys (*n *= 15)	Girls (*n *= 14)
Endurance time (minutes) (mean (standard deviation))		9.0 (1.2)*	9.2 (1.8)
Less than -2 SD, abnormal (*n *(%))	1 (3.4%)	0	1
-1 SD to -2 SD, suspect (*n *(%))	8 (27.6%)	7	1
Greater than -1 SD, normal (*n *(%))	20 (69.0%)	8	12

Score according to Cumming and colleagues [21]. *t test: significant, *P *< 0.001.

**Table 6:** Neuropsychological outcome

	Total group (*n *= 82)
Intelligence quotient (*n *= 79)	100.5 (19.7)
70–85	4 (5.1%)
51–70	3 (3.9%)
≤50	4 (5.1%)
Expressive language (*n *= 78)	
Grammar	104.2^a ^(17.9)
70–85	11 (14.1%)
51–70	2 (2.6%)
≤50	--
Vocabulary	103.2 (19.6)
70–85	6 (7.8%)
51–70	5 (6.5%)
≤50	2 (2.6%)
Receptive language (*n *= 81)	104.3^a ^(15.3)
70–85	1 (1.2%)
51–70	5 (6.1%)
≤50	--
Visual – motor integration (*n *= 28)	96.6 (13.7)
70–85	7 (25%)
51–70	--
≤50	--

Data presented as the mean (standard deviation) or as *n *(% of total number). ^a^Significant difference (*P *< 0.05) from the Dutch population norm.

**Table 7:** Child Behaviour Checklist (*n *= 86)

Total problem score	
<60	72 (83.5%)
60–63	5 (5.9%)
>63	9 (10.5%)
Internal problem score	
<60	72 (83.7%)
60–63	7 (8.1%)
>63	7 (8.1%)
External problem score	
<60	76 (88.4%)
60–63	6 (7.0%)
>63	4 (4.7%)

Data presented as number of patients (%). The internal scale includes withdrawn behaviour, somatic complaints without physical cause, and anxious-depressive feelings. The external scale includes aggressive and delinquent behaviour. Scores ≥60 but <63 are in the borderline range. Scores ≥63 are in the clinical range.
